# Association Between Postpartum Depression Symptoms of Primiparas and Uterine Recovery, Sleep Quality and Postpartum Stress

**DOI:** 10.62641/aep.v54i1.2049

**Published:** 2026-02-15

**Authors:** Hui Gao, Xiao Yang

**Affiliations:** ^1^Department of Obstetrics Nursing, West China Second University Hospital, Sichuan University, 610041 Chengdu, Sichuan, China; ^2^Key Laboratory of Birth Defects and Related Diseases of Women and Children, Sichuan University, Ministry of Education, 610041 Chengdu, Sichuan, China

**Keywords:** postpartum depression, primiparity, uterus, sleep quality, stress

## Abstract

**Background:**

To investigate the relationship between postpartum depression (PPD) symptoms in primiparous women and uterine recovery, sleep quality and postpartum stress.

**Methods:**

This retrospective study enrolled 194 postpartum women who underwent 42-day postpartum examinations in our hospital from February 2024 to February 2025. General demographic information, uterine recovery (including uterine fundal height decline, lochia volume and pelvic floor muscle recovery), sleep quality (determined using the Pittsburgh Sleep Quality Index [PSQI]) and postnatal stress levels (measured using the Maternal Postpartum Stress Scale [MPSS]) were collected through the electronic medical record system. PPD was assessed using the Edinburgh Postnatal Depression Scale (EPDS). Mothers with an EPDS score ≥10 were classified into the PPD group, and those with <10 were classified into the non-PPD group. Univariate and multivariate logistic regression analyses were performed to assess the factors influencing PPD.

**Results:**

A total of 194 primiparous women were included, 56 in the PPD group and 138 in the non-PPD group. The PPD group showed significantly higher percentage of high school education and below, discordant relationship with husband, lack of regular maternity check-ups, illness during pregnancy and poor health of newborn than the non-PPD group (*p* < 0.05). PSQI (*p* = 0.001) and MPSS (*p* < 0.001) scores were significantly higher in the PPD group than in the non-PPD group. In terms of uterine recovery, the PPD group had a significantly lower lochia volume at 24 and 48 h postpartum than the non-PPD group (*p* < 0.001) and poorer recovery of pelvic floor muscle strength (*p* < 0.05). Logistic regression analysis showed that high school education or less, illness during pregnancy, PSQI and MPSS scores were independent risk factors for PPD, whereas higher lochia volume at 24 and 48 h postpartum and pelvic floor muscle strength ≥grade III were protective factors.

**Conclusions:**

This study identified several specific independent risk factors (lower education level, illness during pregnancy, poor sleep quality and high postpartum stress) and protective factors (adequate lochia volume and good pelvic floor muscle recovery) for PPD. Clinical practice should strengthen the identification and intervention of high-risk groups and pay attention to the integrated management of postpartum physiological recovery and mental health.

## Introduction

Postpartum depression (PPD) refers to a common mental health disorder in women after childbirth [1]. This condition is characterised by persistent low mood, loss of interest and anxiety [2]. According to the global burden of disease estimate, the global incidence rate of PPD is approximately 17.22% [3]. PPD not only seriously affects maternal physical and mental health and the quality of life but may also have long-term adverse effects on infant growth and development, parent–child interaction and family functioning [4]. The risk of PPD in primiparous women requires extra attention due to factors, such as the lack of childcare experience and difficulties in adapting to role changes. With the adjustment of reproductive policy and the increase in social concern for women’s health, in-depth investigation of factors affecting PPD in primiparous women is of great clinical and public health significance for early prevention and intervention.

The assessment of uterine recovery typically encompasses multiple dimensions [5]. Uterine fundal height descent denotes a classical and straightforward clinical measure for tracking the pace of uterine involution; a slow descent may indicate subinvolution, which is associated with discomfort and potential infection [6]. Lochia volume, particularly in the immediate postpartum period, serves as a critical indicator of endometrial shedding and regeneration. Abnormally scant or prolonged lochia can signal retained products of conception or infection, both of which are sources of physical pain and psychological distress [7]. Furthermore, the childbirth process poses a major trauma to the pelvic floor. Pelvic floor muscle strength is increasingly recognised as a key component of postpartum recovery, as its impairment is directly linked to urinary incontinence and sexual dysfunction, which severely affect a woman’s quality of life, self-esteem and psychological well-being [8]. A close association exists between postnatal physical discomfort and negative emotions, and the discomfort caused by poor uterine recovery may interfere with a woman’s daily life and increase her psychological stress, which in turn may induce depressive symptoms [9]. The quality of sleep is another key factor that affects the physical and mental health of postpartum women [10]. Factors such as fluctuating hormone levels after childbirth, the need for breastfeeding and nighttime care for newborns make sleep disruption a common problem for postpartum women [11]. Sleep disorders are an important risk factor for PPD [12]. Postnatal stress refers to the psychological pressure that arises in the postpartum period due to a variety of factors, such as changes in maternal roles, adjustments in family relationships and childcare responsibilities [13]. Postpartum stress serves as an important psychosocial factor in the development of PPD [14]. However, systematic research on the quantitative relationship between indicators of uterine recovery, sleep quality and postpartum stress and PPD is still lacking, and the underlying mechanisms of action have not been fully clarified.

This study aimed to further explore the factors influencing PPD and its related mechanisms by investigating postpartum depressive symptoms in primiparous women and their association with uterine recovery, sleep quality and postpartum stress. Through this study, we expect to provide new bases and ideas for the early identification and intervention of PPD and provide scientific support for the improvement of maternal physical and mental health and mother–infant relationship. Meanwhile, this work will also provide reference for clinicians in postnatal health care, helping them to focus more on the physical and mental health of mothers, identify and deal with PPD in a timely manner and improve the quality of life of mothers and the well-being of their families.

## Materials and Methods

### Study Population

We conducted a retrospective study that included parturients who underwent 42-day postpartum check-ups from February 2024 to February 2025 at the obstetrics clinic of the West China Second University Hospital, Sichuan University. A total of 194 study participants were finally included. The study received approval from the Medical Research Ethics Committee of the West China Second University Hospital, Sichuan University (Ethics Approval Number: 2025-068) and strictly adhered to the medical ethical principles of the Declaration of Helsinki. All patients gave informed consent.

### Sample Size Calculation

According to a cross-sectional study of the Chinese population, 20.2% of postpartum women have symptoms of PPD [15]. Therefore, this study adopted 20% as the estimated value for the expected prevalence of PPD to ensure a sufficient sample size representing the disease burden in the target population. Another meta-analysis involving 12,614 pregnant women indicated that sleep disorders were significantly associated with the risk of PPD, with a combined effect size of odds ratio (OR) = 2.36 [16]. The required sample size was calculated using G*Power (version 3.1.9.7; Heinrich-Heine-Universität Düsseldorf; Düsseldorf, Germany). The significance level (α) was set at 0.05 and the test power (1-β) at 0.80. The calculation results show that the total sample size should be at least 155 cases. A total of 194 patient data were analysed in this study, which met the sample size requirements.

### Inclusion and Exclusion Criteria

The inclusion criteria were as follows: (1) singleton pregnancy and first delivery; (2) aged 18–40 years old; (3) visiting the obstetrics outpatient clinic of our hospital for routine checkups on time 42 days after delivery; (4) having normal communication skills and being able to understand and complete the questionnaire independently.

The exclusion criteria included the following: (1) psychiatric disorders (e.g., depression, anxiety disorders, schizophrenia, etc.) diagnosed before pregnancy; (2) serious postnatal complications (e.g., postpartum haemorrhage, puerperal infections, eclampsia, etc.) and are still under therapeutic observation; (3) serious hearing, visual or speech disorders and inability to fill in the scale.

### Data Collection

Data collection was completed by uniformly trained clinicians. The data on research subjects were extracted by the researchers from the hospital’s electronic medical record system; the information included maternal age, education level, couple relationship, monthly household income, whether the pregnancy was unplanned, regularity of antenatal check-ups, presence of pregnancy diseases, mode of delivery, postpartum haemorrhage, newborn’s health status, newborn’s gender and mode of feeding. The height of the uterine fundus descent and amount of lochia at 24 and 48 h after delivery were also collected. The health status of the newborn was assessed at the 42-day postpartum check-up: (1) Good health: A newborn who was full-term (gestational age ≥37 weeks), with a birth weight appropriate for gestational age and without any diagnosed major congenital anomalies, severe neonatal diseases (such as severe neonatal asphyxia, sepsis or need for neonatal intensive care unit admission) or ongoing significant medical concerns at the time of the check-up. (2) Poor health: A newborn who was preterm (gestational age <37 weeks), had low birth weight, and/or was diagnosed with any major congenital anomaly, severe neonatal disease or presented with any significant ongoing medical issue requiring follow-up or treatment. In addition, maternal pelvic muscle recovery was assessed by the obstetrician at the 42-day outpatient visit on the basis of the modified Oxford scale grading system [17]. This system grades pelvic floor muscle strength on a 6-point scale (0 to 5) as follows: 0 = no contraction; 1 = flicker (a minor muscle twitch); 2 = weak contraction (partial muscle compression without resistance); 3 = moderate contraction (complete muscle compression with slight resistance); 4 = good contraction (complete compression against moderate resistance); 5 = strong contraction (complete compression against strong resistance). A high grade indicates desirable recovery of pelvic floor muscle strength [18].

### Survey Methodology

All the clinicians and researchers who participated in the questionnaire survey received professional training, with emphasis on the use of gentle, neutral and noncritical language when asking questions and avoidance of direct or sensitive questions that may cause discomfort to the parturients. In regard to sensitive issues related to postpartum stress, we adopted a step-by-step guiding approach, starting with general problems and gradually transitioning to specific ones, which provided new mothers with sufficient time to adapt and think. If during the investigation, the parturient showed emotional fluctuations or discomfort, the investigators were to immediately suspend the investigation, provide necessary psychological support and refer the patient to a professional psychological counsellor if necessary. At the beginning of the questionnaire, we clearly informed the parturients of the anonymity and confidentiality of the survey to ensure that they could answer the questions with peace of mind and reduce the psychological pressure caused by concerns about privacy leakage.

### Survey Scale

This hospital prioritises the mental wellbeing of maternity patients. Routine mental health questionnaires were administered to all maternity patients. The survey results were routinely incorporated into individual medical records, and the information from the scales were extracted for this research.

The presence of postpartum depressive symptoms was assessed using the Edinburgh Postnatal Depression Scale (EPDS) [19]. The EPDS is a widely used and validated screening tool for the identification of women at high risk for PPD in research and clinical settings, particularly in obstetric populations [19]. Although a formal clinical diagnosis of major depressive disorder requires a comprehensive evaluation based on standardised diagnostic criteria (e.g., Diagnostic and Statistical Manual of Mental Illnesses-5), a cutoff score of ≥10 on the EPDS has good sensitivity and specificity for identifying probable cases of PPD in community and obstetric samples [20, 21]. The EPDS scale consists of 10 items and adopts a 4-level scoring standard (0–3 points), with a score range of 0–30 points. The higher the EPDS score is, the greater the possibility of the patient having depressive symptoms. Those with EPDS scores <10 points were allotted into the non-PPD group, and those with scores ≥10 points were included in the PPD group. In this study, the Cronbach’s alpha coefficient of the EPDS scale was 0.89.

The postpartum sleep status of the research subjects was evaluated using the Pittsburgh sleep quality index (PSQI) [22]. The PSQI scale consists of 19 self-rated questions used to generate scores across seven components: subjective sleep quality, sleep latency, sleep duration, habitual sleep efficiency, sleep disturbances, use of sleeping medication and daytime dysfunction. The PSQI scale adopts a four-level scoring standard (0–3 points), with a score range of 0–21 points. The higher the PSQI score, the poorer the sleep quality. In this study, the Cronbach’s alpha coefficient of the PSQI scale was 0.81.

The postpartum stress level was evaluated using the Maternal Postpartum Stress Scale (MPSS) [23]. The MPSS scale consists of 22 items covering four dimensions: personal needs, infant care, physical changes and sexual behaviour. The MPSS scale adopts a 5-level scoring standard (0–4 points), with a total score ranging from 0 to 88 points. The higher the total score of MPSS, the higher the postpartum stress level. In this study, the MPSS scale had a Cronbach’s alpha coefficient of 0.87.

### Statistical Analysis

Data were analysed using SPSS (Version 27.0; IBM Corp.; Armonk, NY, USA) statistical software. Measurements are expressed as mean ± standard deviation ($\bar{x}$ ± s), where normal distribution was confirmed by the Shapiro–Wilk test, and comparisons between groups were achieved using independent sample *t*-test. Non-normally distributed data were expressed as medians, and the Mann–Whitney U test was used for between-group comparisons. Count data were expressed as the number of cases (percentage) [n (%)], and comparisons between groups were performed using the χ^2^ test or Fisher’s exact probability method (when the theoretical frequency was <5). Independent risk factors were assessed using multifactorial logistic regression analysis, and (ORs) and their 95% confidence intervals (CIs) were calculated. Variables with *p* < 0.05 in univariate logistic regression were included in the multivariate logistic regression model. Multivariate logistic regression analysis was performed using the stepwise regression method (screening criterion: *p* < 0.05). The variance inflation factor (VIF) was used to evaluate the collinearity among variables in the multivariate logistic regression model. VIF <5 was considered to indicate no collinearity among variables. The receiver operating characteristic (ROC) curve and its area under the curve (AUC) were used to assess the discrimination of the logistic model, and the calibration curve and Hosmer–Lemeshow test were used to evaluate the calibration. All the tests were bilateral, and *p* < 0.05 was considered statistically significant.

## Results

### General Information of the Participants

A total of 194 primiparous women, 56 in the PPD group and 138 in the non-PPD group, were included in this study. No significant difference was observed in the distribution of the two groups in terms of age, monthly household income, unplanned pregnancy, mode of delivery, postpartum haemorrhage, sex of the newborns and feeding methods (*p* > 0.05) (Table 1). In the PPD group, the proportion of those with high school or lower education, disharmony with husband, no regular prenatal checkups and the presence of illnesses during pregnancy were significantly higher than that in the non-PPD group. In addition, the PPD group had a higher proportion of newborns with poor health. The PPD group also displayed significantly higher PSQI (9.00 vs. 7.00, *p* = 0.001) and MPSS (18.41 vs. 15.37, *p* < 0.001) scores than the non-PPD group.

**Table 1.** **Comparison of general information between PPD and non-PPD groups**.

Variables		Total (n = 194)	Non-PPD group (n = 138)	PPD group (n = 56)	Statistic	*p*
Age, n (%)					χ^2^ = 0.74	0.390
	<25	96 (49.48)	71 (51.45)	25 (44.64)		
	≥25	98 (50.52)	67 (48.55)	31 (55.36)		
Educational level, n (%)					χ^2^ = 6.63	0.010
	Specialist and above	111 (57.22)	87 (63.04)	24 (42.86)		
	High school and below	83 (42.78)	51 (36.96)	32 (57.14)		
Marital relationship, n (%)					χ^2^ = 5.68	0.017
	Harmony	141 (72.68)	107 (77.54)	34 (60.71)		
	Disharmony	53 (27.32)	31 (22.46)	22 (39.29)		
Monthly household income (CNY)^a^, n (%)					χ^2^ = 1.46	0.226
	≥5000	156 (80.41)	114 (82.61)	42 (75.00)		
	<5000	38 (19.59)	24 (17.39)	14 (25.00)		
Unintended pregnancy, n (%)					χ^2^ = 0.79	0.375
	No	163 (84.02)	118 (85.51)	45 (80.36)		
	Yes	31 (15.98)	20 (14.49)	11 (19.64)		
Regular prenatal check-ups, n (%)					χ^2^ = 5.24	0.022
	No	60 (30.93)	36 (26.09)	24 (42.86)		
	Yes	134 (69.07)	102 (73.91)	32 (57.14)		
Illness during pregnancy, n (%)					χ^2^ = 7.25	0.007
	No	158 (81.44)	119 (86.23)	39 (69.64)		
	Yes	36 (18.56)	19 (13.77)	17 (30.36)		
Mode of delivery, n (%)					χ^2^ = 2.52	0.112
	Vaginal delivery	83 (42.78)	64 (46.38)	19 (33.93)		
	Caesarean section	111 (57.22)	74 (53.62)	37 (66.07)		
Postpartum haemorrhage, n (%)					χ^2^ = 0.81	0.369
	No	117 (60.31)	86 (62.32)	31 (55.36)		
	Yes	77 (39.69)	52 (37.68)	25 (44.64)		
Health status of newborns, n (%)					χ^2^ = 5.68	0.017
	Good health	141 (72.68)	107 (77.54)	34 (60.71)		
	Poor health	53 (27.32)	31 (22.46)	22 (39.29)		
Neonatal gender, n (%)					χ^2^ = 2.10	0.147
	Female infant	95 (48.97)	63 (45.65)	32 (57.14)		
	Male infant	99 (51.03)	75 (54.35)	24 (42.86)		
Feeding methods, n (%)					χ^2^ = 1.98	0.159
	Breastfeeding	81 (41.75)	62 (44.93)	19 (33.93)		
	Artificial feeding	113 (58.25)	76 (55.07)	37 (66.07)		
PSQI score, M (Q_1_, Q_3_)		7.00 (6.00, 9.00)	7.00 (6.00, 9.00)	9.00 (7.00, 11.00)	Z = –3.21	0.001
MPSS score, Mean ± SD		16.25 ± 5.49	15.37 ± 5.19	18.41 ± 5.65	t = –3.61	<0.001

^a^Exchange rate (approximate at time of study): 1 USD ≈ 7.04 CNY. Abbreviation: PPD, postpartum depressive; SD, standard deviation; M, median; Q_1_, 1st quartile; Q_3_, 3rd quartile; PSQI, Pittsburgh Sleep Quality Index; MPSS, Maternal Postpartum Stress Scale.

### Comparison of Uterine Recovery Effects

No significant difference was noticed in the height of uterine fundal descent between the PPD and non-PPD groups at 24 and 48 h postpartum (*p* > 0.05) (Table 2). However, the lochia volume in the PPD group was significantly less than that in the non-PPD group at 24 (*p* < 0.001) and 48 h (*p* < 0.001) postpartum.

**Table 2.** **Comparison of uterine recovery effects**.

Grouping	n	Uterine fundal height decline (mm)		Lochia volume (mL)	
		24 h postpartum	48 h postpartum	24 h postpartum	48 h postpartum
PPD group	56	8.12 ± 1.13	11.47 ± 1.34	204.35 ± 16.47	44.31 ± 5.17
Non-PPD group	138	8.37 ± 1.26	11.82 ± 1.37	214.62 ± 18.53	49.24 ± 5.52
t value		1.35	1.58	3.61	5.73
*p* value		0.180	0.115	<0.001	<0.001

Abbreviation: PPD, postpartum depressive.

### Comparison of Pelvic Floor Muscle Strength Recovery

At 6 weeks postpartum, the PPD group exhibited a poorer pelvic floor muscle strength recovery, as evidenced by significantly higher proportions of grades I (*p* = 0.033) and II (*p* = 0.028) muscle strength than in the non-PPD group, whereas the proportion of those with muscle strength ≥grade III (*p* < 0.001) was significantly lower (Table 3).

**Table 3.** **Pelvic floor muscle strength at 42 days postpartum**.

Grouping	n	Muscle strength grade		
		Grade I	Grade II	≥Grade III
PPD group	56	11	29	16
Non-PPD group	138	12	48	78
t value		4.57	4.81	12.46
*p* value		0.033	0.028	<0.001

Abbreviation: PPD, postpartum depression.

### Univariate Logistic Regression Analysis of PPD

Univariate and subsequent multivariate logistic regression analyses were performed to identify factors independently associated with PPD. The dependent variable was the presence of PPD, defined as an EPDS score ≥10. Independent variables included all demographic, obstetric and postnatal characteristics listed in Table 1 and uterine recovery parameters (uterine fundal height decline and lochia volume) and pelvic floor muscle strength. Univariate logistic regression analysis showed that high school and below literacy (*p* = 0.011), disharmony with husband (*p* = 0.019), illness during pregnancy (*p* = 0.008), newborns in poor health (*p* = 0.019) and higher PSQI (*p* = 0.002) and MPSS scores (*p* < 0.001) were significantly associated with a high risk of PPD (Table 4). In addition, having regular prenatal check-ups (*p* = 0.023), high lochia volume at 24 (*p* = 0.001) and 48 h (*p* < 0.001) and pelvic floor muscle strength ≥grade III (*p* = 0.003) were associated with a reduced risk of PPD reduction.

**Table 4.** **Univariate Logistic regression**.

Variables		β	SE	Z	*p*	OR (95% CI)	Assignment
Age							
	<25					1.00 (Reference)	0
	≥25	0.27	0.32	0.86	0.391	1.31 (0.70–2.45)	1
Educational level							
	Specialist and above					1.00 (Reference)	0
	High school and below	0.82	0.32	2.55	0.011	2.27 (1.21–4.28)	1
Marital relationship							
	Harmony					1.00 (Reference)	0
	Disharmony	0.80	0.34	2.35	0.019	2.23 (1.14–4.36)	1
Monthly household income (CNY)^a^							
	≥5000					1.00 (Reference)	0
	<5000	0.46	0.38	1.20	0.229	1.58 (0.75–3.35)	1
Unintended pregnancy							
	No					1.00 (Reference)	0
	Yes	0.37	0.41	0.88	0.377	1.44 (0.64–3.25)	1
Regular prenatal check-ups							
	No					1.00 (Reference)	0
	Yes	–0.75	0.33	–2.27	0.023	0.47 (0.25–0.90)	1
Illness during pregnancy							
	No					1.00 (Reference)	0
	Yes	1.00	0.38	2.63	0.008	2.73 (1.29–5.77)	1
Mode of delivery							
	Vaginal delivery					1.00 (Reference)	0
	Caesarean section	0.52	0.33	1.58	0.114	1.68 (0.88–3.21)	1
Postpartum haemorrhage							
	No					1.00 (Reference)	0
	Yes	0.29	0.32	0.90	0.370	1.33 (0.71–2.50)	1
Health status of newborns							
	Good health					1.00 (Reference)	0
	Poor health	0.80	0.34	2.35	0.019	2.23 (1.14–4.36)	1
Neonatal gender							
	Female infant					1.00 (Reference)	0
	Male infant	–0.46	0.32	–1.45	0.148	0.63 (0.34–1.18)	1
Feeding methods							
	Breastfeeding					1.00 (Reference)	0
	Artificial feeding	0.46	0.33	1.40	0.161	1.59 (0.83–3.03)	1
24 h uterine fundal height decline		–0.18	0.13	–1.34	0.180	0.84 (0.65–1.08)	
48 h uterine fundal height decline		–0.19	0.12	–1.57	0.116	0.83 (0.66–1.05)	
24 h lochia volume		–0.03	0.01	–3.37	<0.001	0.97 (0.95–0.99)	
48 h lochia volume		–0.17	0.03	–4.93	<0.001	0.84 (0.79–0.90)	
PSQI score		0.18	0.06	3.15	0.002	1.20 (1.07–1.35)	
MPSS score		0.11	0.03	3.39	<0.001	1.11 (1.05–1.19)	
Pelvic floor muscle strength							
	Grade I					1.00 (Reference)	0
	Grade II	–0.42	0.48	–0.87	0.384	0.66 (0.26–1.69)	1
	≥Grade III	–1.50	0.50	–3.00	0.003	0.22 (0.08–0.60)	2

^a^Exchange rate (approximate at time of study): 1 USD ≈ 7.04 CNY. Abbreviation: OR, odds ratio; CI, confidence interval; SE, standard error; PSQI, Pittsburgh Sleep Quality Index; MPSS, Maternal Postpartum Stress Scale.

### Multivariate Logistic Regression Analysis of PPD

Multivariate logistic regression analysis showed that high school and below literacy (OR = 2.33, 95% CI: 1.05 to 5.18, *p* = 0.039), illness during pregnancy (OR = 2.91, 95% CI: 1.09 to 7.78, *p* = 0.033), and high PSQI (OR = 1.20, 95% CI: 1.04 to 1.39, *p* = 0.013) and MPSS scores (OR = 1.11, 95% CI: 1.03 to 1.20, *p* = 0.005) were independent risk factors for PPD (Table 5). By contrast, a higher lochia volume at 24 (OR = 0.97, 95% CI: 0.95 to 0.99, *p* = 0.016) and 48 h (OR = 0.82, 95% CI: 0.75 to 0.89, *p* < 0.001) and pelvic floor muscle strength ≥grade III (OR = 0.25, 95% CI: 0.08 to 0.80, *p* = 0.020) served as protective factors.

**Table 5.** **Multivariate logistic regression**.

Variables		β	SE	Z	*p*	OR (95% CI)	VIF
Educational level							
	Specialist and above					1.00 (Reference)	
	High school and below	0.84	0.41	2.07	0.039	2.33 (1.05–5.18)	1.01
Illness during pregnancy							
	No					1.00 (Reference)	
	Yes	1.07	0.50	2.13	0.033	2.91 (1.09–7.78)	1.03
24 h lochia volume		–0.03	0.01	–2.41	0.016	0.97 (0.95–0.99)	1.02
48 h lochia volume		–0.20	0.05	–4.41	<0.001	0.82 (0.75–0.89)	1.03
PSQI score		0.19	0.07	2.48	0.013	1.20 (1.04–1.39)	1.02
MPSS score		0.11	0.04	2.79	0.005	1.11 (1.03–1.20)	1.03
Pelvic floor muscle strength							
	Grade I					1.00 (Reference)	
	Grade II	–0.02	0.59	–0.03	0.973	0.98 (0.31–3.10)	
	≥Grade III	–1.40	0.60	–2.33	0.020	0.25 (0.08–0.80)	1.01

Abbreviation: OR, odds ratio; CI, confidence interval; SE, standard error; VIF, variance inflation factor; PSQI, Pittsburgh Sleep Quality Index; MPSS, Maternal Postpartum Stress Scale.

### Evaluation of Multifactor Models

**Fig. 1.**
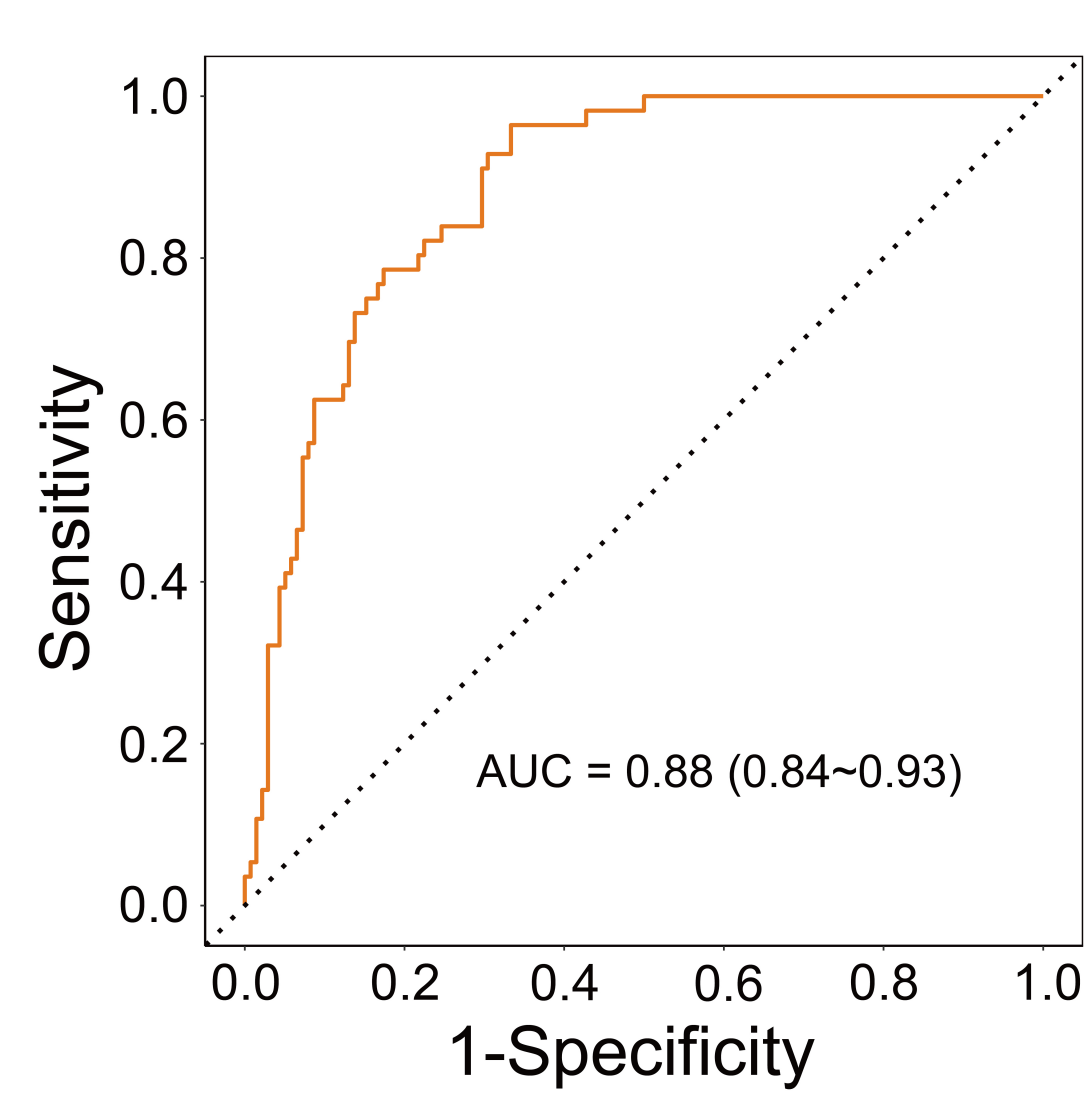
**ROC curve based on the logistic regression model**. Abbreviation: ROC, receiver operating characteristic; AUC, area under the curve.

**Fig. 2.**
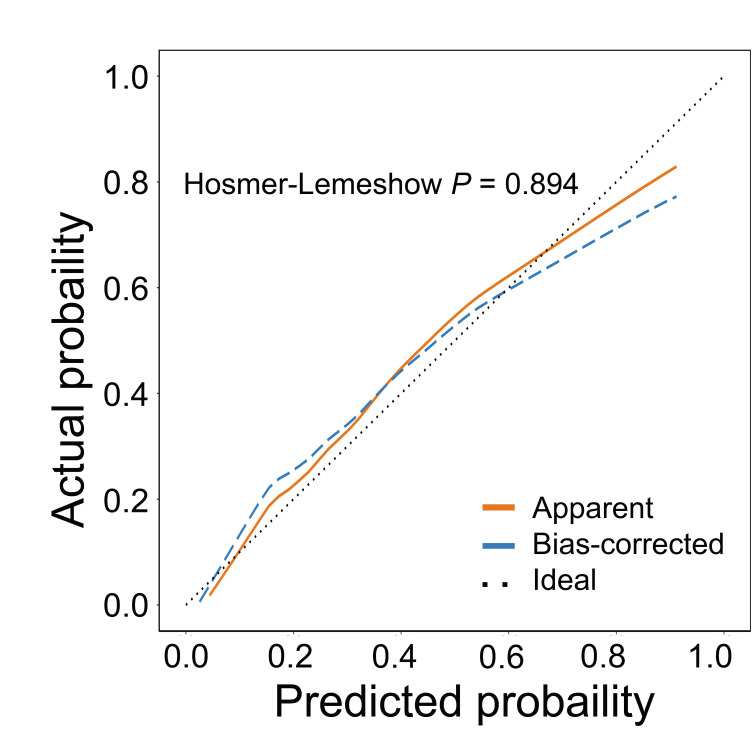
**Calibration curve based on the logistic regression model**.

The VIF values of all variables in the multivariate logistic regression model were less than 5, which indicates that no collinearity was observed among the variables (Table 5). Fig. 1 shows the ROC curve based on the multivariate logistic regression model. The AUC of the multivariate logistic regression model was 0.88, which implies that this model has a high discriminatory capability for the PPD population. Fig. 2 illustrates the calibration curve of this model. No significant difference was recorded between the observed and predicted values of this model, which indicates that the model had a good fit.

## Discussion

PPD is a common mental disorder in the postpartum period, and it seriously affects maternal physical and mental health and the mother–infant relationship [1]. This study analysed the association of PPD with uterine recovery, sleep quality and postpartum stress through a retrospective analysis of 194 primiparous women. The results show that the depressed group differed significantly from the nondepressed group in terms of literacy, couple relationship, regularity of antenatal checkups, the presence of illnesses during pregnancy and health status of the newborn and had higher scores on the PSQI and MPSS. Meanwhile, the depressed group had less lochia volume and poorer recovery of pelvic floor muscle strength. Logistic regression analysis showed that sleep quality, postnatal stress, uterine recovery indicators (lochia volume and pelvic floor muscle strength) and sociodemographic factors independently influenced postnatal depression.

Firstly, the results of the study show that some demographic characteristics were significantly associated with PPD. In this work, the risk of PPD among primiparous women with a high school education or less was 2.33 times higher than that among those with a specialist education or above. Jimènez-Barragan *et al*. [24] also reported through a prospective study that educational level is one of the risk factors for depression during pregnancy. This condition may be related to the limited access of mothers with low educational attainment to scientific childcare knowledge and their weaker psychological adjustment ability. The low-education group is more likely to be frustrated by neonatal care problems, and they are less aware of PPDand lack the awareness to actively seek professional help [25]. The risk of depression among parturients with disharmonious marital relationships is significantly increased, which suggests that having a family support system is crucial for postpartum mental health. Fang *et al*. [26] also stated through a study of 151 parturients that marital disharmony is an independent risk factor for PPD, which is consistent with the results of this study. A close and harmonious marital relationship can provide emotional support and relieve the parenting pressure of new mothers. However, tense relationships may lead to emotional isolation of new mothers and intensify negative emotions [27]. In addition, a history of illness during pregnancy is significantly associated with PPD, which may be caused by illnesses during pregnancy that increased the mother’s concern for her own health and that of the foetus, leading to prenatal anxiety that carries over into the postpartum period [28]. Mothers with poor neonatal health are at high risk of PPD, which may be associated with infant and child health problems that increase the burden of care on the mother and trigger anxiety [26]. Regular antenatal check-ups are a protective factor, probably because they help mothers to obtain timely health guidance and reduce unknown fears about labour and the postnatal period, reflecting the importance of perinatal care in mental health interventions.

In the present study, although no significant difference was observed in the height of uterine fundal descent between the depressed and non-depressed groups, the former had significantly less lochia volume and a lower proportion of pelvic floor muscle strength ≥grade III at 24 and 48 h postpartum. A reduced lochia volume may reflect poor uterine contraction or delayed endothelial repair. We speculate that this physiological abnormality can be linked to mental health through several potential pathways: firstly, by directly contributing to physical discomfort, which may correlate with reduced maternal quality of life; secondly, through its potential association with inflammatory processes, as pro-inflammatory cytokines (e.g., interleukin-6, tumour necrosis factor-alpha) correlate with depressive states [29, 30]. However, the cross-sectional nature of our data cannot rule out the possibility of reverse causality, whereby PPD symptoms may influence physical recovery. In addition, the risk of PPD is high in women with poor recovery of pelvic floor muscle strength (grade I or II). Impaired pelvic floor muscle function may lead to urinary incontinence or sexual dysfunction, which further aggravates the psychological burden [8, 31]. By contrast, the protective effect of pelvic floor muscle strength ≥grade III may be associated with high maternal satisfaction with recovery, which helps to enhance postnatal self-confidence and emotional stability of mothers.

PSQI and MPSS scores were significantly higher in the depressed group than in the non-depressed group, and multifactorial analyses showed both to be independent risk factors. Baattaiah *et al*. [32] reported through a cross-sectional study the significantly positive correlation of PSQI score with the EPDS score, which means that mothers with poor sleep quality has a high risk of developing PPD. Postpartum sleep disturbances may be related to factors, such as changes in hormone levels, the need to breastfeed at night and care of the newborn, which together contribute to disrupted maternal sleep and reduced sleep quality [33]. Chronic sleep deprivation has been theorised to interfere with normal brain function and emotion regulation, which can increase the vulnerability to depression [29, 30]. Nevertheless, pre-existing depressive symptoms contribute to the poor sleep quality observed. Postnatal stress may arise from role changes, adjustments in family relationships and childcare responsibilities [34]. These stresses, if not effectively relieved, may further aggravate the maternal psychological burden and lead to depression [35].

Based on our findings, we propose a more integrated and proactive clinical approach. Firstly, for early identification of high-risk primiparas, routine postpartum care at the 42-day check-up should be expanded to include not only physical examination but also brief, standardised screenings for PPD (e.g., using the EPDS), sleep quality (e.g., using 1–2 key questions from the PSQI scale) and postpartum stress. Crucially, clinicians should be alerted that women presenting with objective signs of suboptimal physiological recovery—specifically, a history of scanty lochia in the immediate postpartum period or poor pelvic floor muscle strength (below Grade III) at the examination—constitute a key high-risk subgroup warranting closer psychological assessment. Secondly, regarding the timing of intervention, our results underscore the importance of the entire early postpartum period. Although the 42-day check-up is a critical window for formal screening, awareness and supportive interventions should begin earlier. Before discharge, mothers—especially those with lower education or a history of pregnancy illness—can receive preemptive education on normative recovery and mental health. Following discharge, community health workers can conduct proactive follow-ups within the first two weeks to assess sleep challenges and stress levels to provide initial support and facilitate referral if needed. This stepped-care model, which integrates physiological indicators with psychosocial screening across the postpartum continuum, can considerably improve the precision and effectiveness of PPD prevention.

This study encountered some limitations. Firstly, its retrospective, cross-sectional design precluded causal inference; thus, we cannot determine whether poor uterine recovery precipitates PPD, or vice versa. Secondly, the sample was modest and recruited from a single tertiary centre, which might have introduced selection bias and limit external validity. Thirdly, we examined the main effects only and did not test potential interactions (e.g., sleep quality × postpartum stress) that can reveal synergistic pathways to PPD. Fourthly, survivorship bias is possible: women with severe depressive symptoms are less likely to attend the 42-day visit, which potentially underestimates PPD prevalence and attenuating risk estimates. Fifthly, uterine involution was assessed clinically (fundal height and lochia volume) rather than by transvaginal ultrasound, which might have reduced sensitivity in detecting subtle subinvolution. Finally, unmeasured confounders—such as inflammatory cytokines or sex-steroid fluctuations—may residualise observed associations. Future prospective, multicentre cohorts incorporating objective imaging and biomarkers are needed to clarify the temporal sequence and biological mechanisms linking physiological recovery with postpartum mental health.

## Conclusions

This study identified psychosocial–physiological risk factors (lower education, illness during pregnancy, poor sleep quality and high postpartum stress) and physiological protective factors (adequate early lochia volume and good pelvic floor muscle recovery) for PPD in primiparous women. The findings underscore the multifactorial nature of PPD and highlight the critical importance of an integrated approach to postpartum care that addresses mental health and physical recovery. Future research should focus on verifying these associations longitudinally and exploring their underlying mechanisms to inform effective prevention and treatment strategies.

## Availability of Data and Materials

The datasets generated and analyzed during the current study are not publicly available due to patient privacy and confidentiality considerations but are available from the corresponding author on reasonable request. Requests for data access should be directed to the corresponding author.
